# Enhanced Energized Dispersive–Guided Extraction Using Doehlert Matrix Optimization and Antioxidant Evaluation of Phenolic Compounds From Herbal Leaves by Using High‐Performance Liquid Chromatography

**DOI:** 10.1002/jssc.70250

**Published:** 2025-09-10

**Authors:** Rosianne P. Silva, Paulo N. A. Santos, Luciana L. Nascimento, Alini T. Fricks, Allan S. Polidoro, Elina B. Caramão

**Affiliations:** ^1^ Programa De Pós‐Graduação em Química Universidade Federal de Sergipe São Cristóvão Sergipe Brazil; ^2^ Rede Nordeste de Biotecnologia Universidade Federal de Sergipe São Cristóvão Sergipe Brazil; ^3^ Departamento De Análise Bromatológica, Programa de Pós‐Graduação em Ciência de Alimentos (PGALI), Faculdade de Farmácia Universidade Federal da Bahia Salvador Brazil; ^4^ Department of Chemistry, Pharmaceutical, and Agricultural Sciences University of Ferrara Ferrara Italy; ^5^ Instituto Nacional de Ciência e Tecnologia Energia e Ambiente (INCT E&A) Salvador Bahia Brazil

**Keywords:** antioxidant activities, herbal leaf extraction, pressurized liquid extraction, total phenolic compounds

## Abstract

Secondary metabolites are important bioactive compounds for diet and medicine. This study optimizes the extraction of hydroethanolic herbal extracts using an EDGE (Energized Dispersive Guided Extraction) system, evaluates their antioxidant capacity, and analyzes correlations among antioxidant activity, total phenolic content, and individual compounds. A Doehlert matrix design was used to optimize extraction, having temperature and time as independent variables, and total phenolic content (mg GAE/g) as the response, quantified via the Folin–Ciocalteu method. Response surface methodology and analysis of variance validated the model. Extracts were characterized by high‐performance liquid chromatography with diode array detector, and antioxidant capacity was assessed using three different assays. Correlation analysis explored potential synergistic effects. Optimal extraction conditions (40% ethanol, 60% water, 165.82°C, 6.03 min) yielded a significant model (*p* < 0.05, *R* ^2^ = 0.99). The mean total phenolic content was 69.18 mg GAE/g (SD = 0.73%). Chromatographic analysis confirmed phenolic acids, highlighting rutin in *Crataegus oxyacantha* and theophylline in *Sorocea bonplandii*. Antioxidant assays indicated a strong presence of active compounds, and correlation analysis suggested synergistic effects among constituents. The optimized extraction method effectively enhanced phenolic compound yield and antioxidant properties. The findings highlight potential synergistic interactions among phenolic constituents, contributing to the functional properties of the herbal extracts.

## 1 Introduction

Medicinal plants are an important source of bioactive metabolites and continue to play a central role in both traditional and modern healthcare systems. According to the World Health Organization (WHO), approximately 80% of the global population relies on herbal medicines for primary healthcare, and nearly 25% of modern pharmaceutical drugs are still plant‐derived [1]. Various species are used in the preparation of herbal infusions, which are known to be rich in polyphenolic antioxidants and other bioactive constituents that contribute to health maintenance and disease prevention [2].

Polyphenolic compounds are key secondary metabolites that influence the sensory, nutritional, and antioxidant properties of plant materials [3]. Due to their ability to neutralize reactive oxygen species (ROS), they have been extensively studied for their therapeutic properties and are widely applied in pharmaceutical, cosmetic, nutraceutical, and food sectors [2].

These compounds have been consistently associated with 60 significant health benefits in humans [2].

The beneficial properties and characteristic flavor of herbal infusions are largely attributed to the presence of polyphenols. Their antioxidant activity arises from the phenolic hydroxyl groups, which are capable of scavenging free radicals (a major factor in oxidative stress) related to diseases such as aging and cancer [3].

Efficient extraction of polyphenolic compounds is essential for reliable quantification and comprehensive chemical characterization of plant materials [4]. Modern extraction techniques have been developed for efficiency, reproducibility, and yield, while minimizing environmental impact. Extraction is a critical step in sample preparation, directly affecting the selectivity and sensitivity of analytical methods. Careful selection of solvents, reagents, and preconcentration steps is essential for effective analyte separation and concentration in the final extract [5].

An ideal extraction method should be rapid, non‐destructive, environmentally friendly, and capable of yielding high‐quality extracts. In response to growing demand, especially in the food and nutraceutical industries, green extraction methods have been increasingly adopted to meet sustainability requirements [9]. Conventional extraction techniques, such as percolation, maceration, hydrodistillation, and the Soxhlet extraction, remain in use due to their simplicity and cost‐effectiveness [6, 7]. However, they typically require large amounts of solvents, extended extraction times, and high processing temperatures, which may degrade thermolabile compounds, including antioxidants [8].

To overcome these limitations, several extraction methods, including solid‐phase extraction (SPE) and liquid–liquid extraction (LLE), have been employed for isolating bioactive compounds from plant materials, often yielding high‐quality extracts.

Among these innovations, the Energized Dispersive Guided Extraction (EDGE) has emerged as a promising automated alternative to traditional extraction methods. This technique integrates dispersive SPE (DSPE) with pressurized liquid extraction (PLE), offering shorter extraction times, greater automation than conventional approaches, more automation than QuEChERS extraction, and simpler operation than other solvent‐based methods [6, 7].

EDGE system utilizes a unique two‐part aluminum vessel known as the Q‐Cup, fitted with a filtration system (Q‐Disc). Samples are loaded into the Q‐Cup and placed into a sealed chamber, where solvent is added from the top (0–30 mL), between the extractor cell and chamber walls (0–10 mL), or both. The chamber walls are then heated (103 25–200°C), allowing the solvent to expand and apply pressure, promoting solvent penetration through the sample and facilitating analyte desorption and recovery [6].

Solvent‐based techniques for extracting phenolic compounds depend on critical variables such as solvent composition, temperature, time, and solid‐to‐liquid ratio [9]. These parameters, together with the plant matrix properties, significantly influence the polyphenolic content of the extract [5]. To optimize extraction performance, statistical tools such as design of experiments (DoE) and chemometric models have proven to be highly effective.

DoE enables the simultaneous evaluation of multiple variables and their interactions, reducing experimental workload and increasing result reliability [9]. Within DoE strategies, response surface methodology (RSM) is particularly valuable, providing comprehensive insights while minimizing the number of required experiments [10].

In this study, EDGE extraction was optimized for obtaining hydroethanolic extracts from medicinal herb leaves. *Cymbopogon citratus* (CC) leaf was selected for initial method development, and the optimized conditions were subsequently applied to infusion samples prepared from *Crataegus oxyacantha* (CO) and *Sorocea bonplandii* (SB). A Doehlert matrix (DD) design was employed to evaluate the effects of extraction time and temperature.

RSM was used to identify optimal conditions for maximizing total phenolic compound (TPC) through mathematical modeling. The chemical profiles of the optimized extracts were characterized by high‐performance liquid chromatography (HPLC)‐photodiode detector array (PDA), and their antioxidant capacities were assessed using 2,2‐diphenyl‐1‐picrylhydrazyl (DPPH), ABTS, and ferric reducing antioxidant power (FRAP) assays.

## 2 Experimental Procedures

### 2.1 Plant Material and Chemicals

Dried leaves of CO, SB (*Sorocea bonplandii*), and CC were purchased from Rocha Saúde Group (Lot GRS10), a specialized herbal products store located in São Paulo, Brazil. According to the supplier, the materials were taxonomically identified at the species level and consist exclusively of aerial parts traditionally used for infusion preparations. Upon receipt, the samples were grounded, sieved (35 mesh), and oven‐dried at 65°C for 24 h. The processed material was stored in amber flasks under dry and dark conditions until analysis.

All solvents were of chromatographic grade and obtained from J. T. Baker. Trolox (6‐hydroxy‐2,5,7,8‐tetramethylchroman‐2‐carboxylic acid), DPPH, ABTS diammonium salt, 2,4,6‐tri(2‐pyridyl)‐*s*‐triazine (TPTZ), Folin–Ciocalteu reagent, anhydrous sodium carbonate, gallic acid, potassium persulfate, iron(III) chloride hexahydrate, and sodium acetate trihydrate were purchased from Merck (Darmstadt, Germany). Standards of caffeic, *p*‐coumaric, ferulic, and cinnamic acids, as well as theophylline and rutin, were obtained from Sigma‐Aldrich (Brazil). Stock standard solutions (10 mg/L) were prepared in high‐purity methanol and diluted with methanol to obtain working solutions.

### 2.2 Optimization Procedure of Extraction (EDGE)

The extraction was optimized using a Doehlert matrix design with temperature (°C) and time (s) as independent variables, whereas total phenolic compound (TPC, mg GAE/g) was the dependent variable, quantified by the Folin–Ciocalteu method (Table S1, supplementary electronic material [SEM]). The kind of extractor solvent, the proportion between ethanol and water, and the mass of sample to be used were optimized in previous work of our group [6, 7]. The extraction protocol, initially optimized using CC leaves and later applied to CO and SB samples, involved processing 500 mg of dried plant material in a Q‐Cup equipped with an S1 Q‐Disc filter set (C9 + G1 + C9). This set is a standard configuration used in the EDGE extraction system, consisting of a sandwich of three filters (C9 + G1 + C9), each with specific filtration and separation properties. This setup is recommended for general applications, ensuring efficient particle retention during extraction.

The extraction employed 20 mL of a 40%:60% ethanol:water solution, followed by a system cleaning step using 10 mL of extraction solvent (180°C, 30 s) between samples.

### 2.3 Total Phenolic Compound (TPC)

Total phenolic content was determined spectrophotometrically using the Folin–Ciocalteu method [6]. A 100 µL aliquot of the extract was mixed with 8.0 mL of ultrapure water, 500 µL of Folin–Ciocalteu reagent, and 5.0 mL of Na_2_CO_3_ solution (7% w/v). After 2 h of incubation at room temperature, the absorbance was measured at 765 nm using a UV–Vis spectrophotometer (Hach DR 5000). Results were expressed as mg of gallic acid equivalents (GAE) per gram of dry sample (mg GAE/g). All measurements were performed in triplicate.

### 2.4 In Vitro Antioxidant Activity Assays

#### 2.4.1 DPPH Radical Scavenging Assay

The radical scavenging activity was determined following the method of Brand‐Williams [6]. A 100 µL aliquot of methanolic extract solutions at varying concentrations was mixed with 3.9 mL of a 0.06 mM DPPH methanolic solution. After 1 h of incubation at 25°C, absorbance was measured at 515 nm, using a UV–Vis spectrophotometer (Hach DR 5000) and a quartz cuvette (1.00 cm path length). Trolox was used as a standard, and methanol was used as a blank. Results were expressed as milligrams of Trolox equivalent (mg TE) per g of dry sample (mg TE/g). Experiments were performed in triplicate.

#### 2.4.2 ABTS Radical Scavenging Assay

The ABTS assay was performed with minor modifications to previously described methods [11]. A 30 µL aliquot of each extract was added to 3.0 mL of ABTS•+ working solution, pre‐adjusted to an absorbance adjusted to 0.710 ± 0.050. After 6 min of reaction at room temperature, absorbance was measured at 734 nm. Results were expressed as mg TE/g. The ABTS•+ solution was prepared by incubating ABTS with potassium persulfate overnight in the dark at 25°C and diluted with ethanol prior to use.

#### 2.4.3 FRAP Radical Scavenging Assay

The FRAP assay was conducted on the basis of the method described by Benzie and Strain [12], which measures the reduction of Fe^3+^ to Fe^2+^. The extracts were diluted to three concentrations. A 90 µL aliquot of each diluted solution was mixed with 270 µL of distilled water and 2.7 mL of FRAP reagent. The mixture was incubated at 37°C for 30 min, and absorbance was measured at 595 nm. Results were expressed as µmol Fe^2+^ per gram of sample.

### 2.5 Liquid Chromatographic Analysis

Phenolic compounds were analyzed by HPLC (Shimadzu, Japan) equipped with a CBM‐20A system controller, CTO‐20A column oven, SIL‐20A autosampler, and SPD‐M20A PDA. Separation was performed on a Luna C18 column (250 mm × 4.6 mm) with a guard column of the same stationary phase. The mobile phase consisted of ultrapure water acidified with 5% acetic acid (A) and methanol (B). The flow rate was 1.0 mL/min, the column temperature was set to 40°C, and the injection volume was 20 µL. The flow rate was 1 mL/min, the column temperature was 40°C, and the injection volume was 20 µL.

Gradient elution was applied as follows: The run started isocratically at 5% solvent B from 0 to 3 min, followed by a linear increase to 40% B over 3–20 min. The composition was held isocratically at 40% B between 20 and 35 min and then increased linearly to 50% B from 35 to 45 min. A final ramp to 95% B was applied over 45–55 min. Re‐equilibration to initial conditions (5% B) was performed from 66 to 70 min, aiming for better separation and resolution of complex mixtures, ensuring accurate identification and quantification of compounds. UV absorbance was monitored from 200 to 400 nm. Quantification of the analytes was achieved at 255 nm for rutin, 275 nm for theophylline, 322 nm for caffeic acid, 310 nm for *p*‐coumaric acid, 240 nm for ferulic acid, and 280 nm for *trans*‐cinnamic acid based on the analytical response of certified standards.

### 2.6 Statistical Analysis

Analysis of variance (ANOVA) and RSM were performed using Design Expert software. ANOVA was used to assess statistical significance between groups, whereas RSM was applied to explore variable interactions and optimize experimental conditions. All statistical analyses were conducted at a 5% significance level to ensure result robustness.

## 3 Results and Discussion

### 3.1 Phenolic Extraction Optimization

#### 3.1.1 Optimization of EDGE Process for Enhanced Content of Bioactive Compounds

To maximize the conditions for the extraction of phenolic compounds from herbs, dry leaves from CC were submitted to EDGE extraction according to Table 1, employing the RSM in conjunction with the Doehlert design. An effective experimental design approach, the Doehlert matrix is particularly suited for process enhancement in analytical chemistry. The independent variables assessed in this study were temperature and extraction time, with the total phenolic compound content as the dependent variable. Temperature was studied at five distinct levels (120°C; 135°C; 150°C; 165°C; 180°C), whereas extraction time was examined at three different levels (4.74; 6.47; 8.20 min).

**TABLE 1:** Coded and real values with SRM responses from the Doehlert design.

Exp.	Temperature (°C) (*x*_1_)	Time (min) (*x*_2_)	TPC (mg GAE/g)
1	180 (1)	6.47 (0)	67.86
2	165 (0.5)	8.20 (0.866)	62.04
3	120 (−1)	6.47 (0)	53.98
4	135 (−0.5)	4.74 (−0.866)	56.67
5	165 (0.5)	4.74 (−0.866)	66.82
6	135 (−0.5)	8.20 (0.866)	58.16
7 (C)	150 (0)	6.47 (0)	69.85
8 (C)	150 (0)	6.47 (0)	69.54
9 (C)	150 (0)	6.47 (0)	68.16

*Note*: TPC: measured in mg of Gallic Acid Equivalent per gram of dried sample.

**FIGURE 1:**
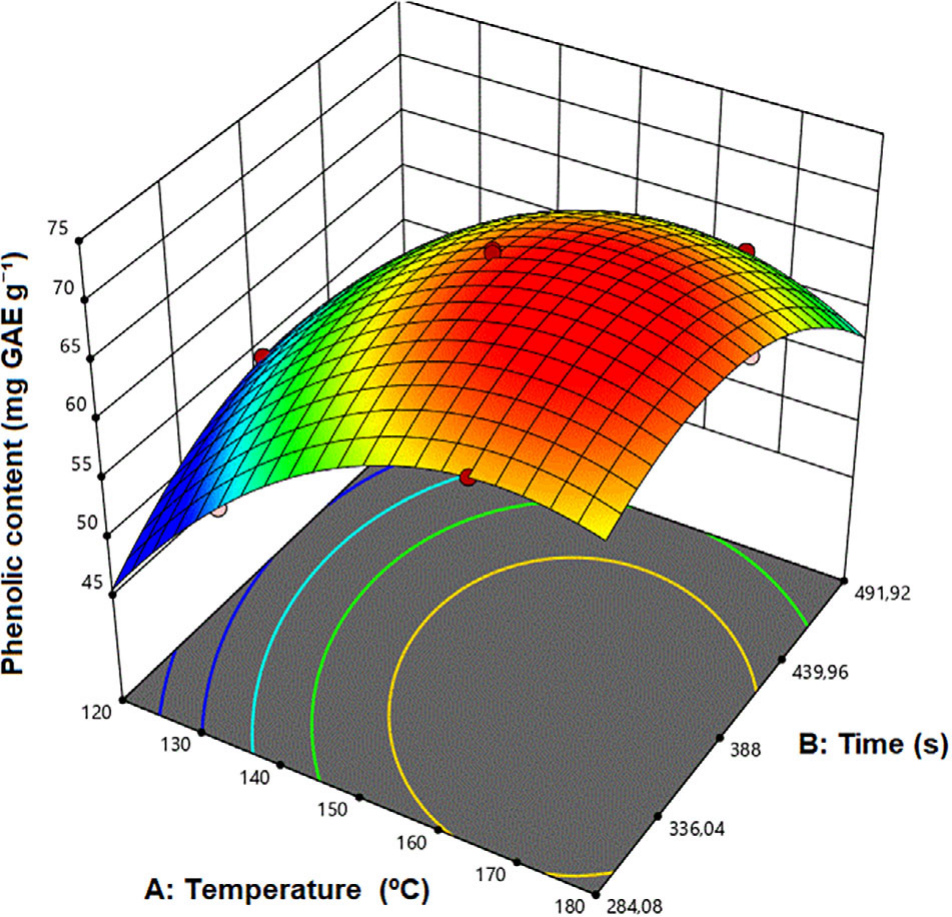
Response surface 3D obtained by the Doehlert design of the experimentally evaluated region.

There are distinct advantages to studying the effects of temperature and extraction time on analytical procedures with this uniform shell design, as it requires fewer experimental points than comparable ones while retaining high predictive power [13]. The matrix generates a uniform distribution of points in the experimental space, arranged in a hexagonal pattern for two variables, where temperature (*x*_1_) and extraction time (*x*_2_) used to investigate different levels. One of its key strengths lies in its rotatable nature and the ability to extend the experimental domain in directions of interest with minimal additional experiments. The design consists of *N* = *k* ^2^ + *k* + 1 experiments, where *k* represents the number of variables (in this case, *k* = 2, resulting in 7 experimental points) [14]. Table 1 presents the results of the analysis of the total phenolic compounds of the extracts obtained through the experimental design applied to the extraction of the lemongrass sample (CC). Figure 1 represents the 3D response surface for the data obtained. The optimal extraction conditions were 6.03 min at a temperature of 165.82°C.

This optimization strategy enables the development of second‐order polynomial models that can effectively describe the response surface and identify optimal conditions with fewer experimental runs compared to traditional designs of DoEs [13, 14]. ANOVA was performed to assess the influence of the independent variables on the response (dependent) variable. The results indicated that the variable extraction temperature (*x*_1_, *p* = 0.0005) and the interaction between temperature and time (*x*_1_ *x*_2_, *p* = 0.0237) are statistically significant (Table S2—SEM). In contrast, extraction time (*x*_2_, *p* = 0.1113) was not significant at a confidence level of 95%.

The computed *F* value for the lack of fit (Table S2) was 0.0038, indicating that the lack of fit was not statistically significant when compared to the pure error, thereby validating the adequacy of the model.

These data supported the calibration of the mathematical model (Equation 1), which was used to predict the response based on the significant variables influencing the extraction process and to estimate the maximum expected response within a 95% confidence interval:

(1) $$\begin{array}{cc} \text{TPC}\;(\text{mg GAE}/g) & =69.18\left(\pm 1.35\right)+6.96\left(\pm 1.35\right)x_{1}\\ & \;-0.8225\left(\pm 1.17\right)x_{2}-3.13\left(\pm 2.35\right)x_{1}x_{2}\\ & \;-8.26\left(\pm 2.13\right)x_{1}^{2}-6.19\left(\pm 1.60\right)x_{2}^{2} \end{array}$$

In this study, TPC was considered the response variable, whereas *x*_1_ (temperature) and *x*_2_ (time) are the independent variables. To assess the predictive adequacy of the proposed model, triplicate extractions were conducted under the optimized conditions. When applied to the lemongrass sample, these conditions yielded an average TPC of 69.18 mg GAE/g, which closely matched the model's predicted value of 70.03 mg GAE/g, representing a deviation of only 1.2%. This result supports the predictive accuracy of the modeling framework.

The robustness of the optimized mathematical model was further confirmed by a high correlation coefficient (*R* ^2^ = 0.99), demonstrating the reliability of the experimental design across the defined experimental domain. This level of precision can be attributed, in part, to the automated nature of the extraction system, which minimizes operational variability and experimental error.

#### 3.1.2 Phenolic Content of Optimal Extracts Obtained by EDGE

Following the establishment of optimal extraction conditions for phenolic compounds in the CC sample, these conditions were subsequently applied to the SB and CO samples. The phenolic compound content was quantified using the Folin–Ciocalteau method, yielding values of 25.92  (± 0.01) and 85.62 (± 0.01) mg GAE/g for the SB and CO samples, respectively.

The lemongrass extract (CC) showed a total phenolic content of 69.65 mg GAE/g, obtained via EDGE using 20 mL of an ethanol–water mixture (40%, v/v). Kouassi et al. [15] reported a TPC of 35.43 mg GAE/g in lemongrass leaves extracted with pure ethanol using Soxhlet. Irfan et al. [16] assessed the phenolic content of hydroethanolic extracts of lemongrass obtained by maceration and sonication with ethanol and water. For maceration, TPC ranged from 36.80 mg GAE/g (50:50, v/v) to 32.90 mg GAE/g (70:30, v/v), whereas sonication yielded a phenolic content of 61.21 and 54.10 mg GAE/g for the same solvent systems.

The EDGE technique is a non‐conventional solid–liquid extraction method performed at elevated temperatures (up to 200°C) and moderate pressures (up to 200 psi). This combination of temperature and pressure enhances extraction efficiency by improving solvent diffusion through reduced viscosity and surface tension. As a result, solvent penetration into the solid matrix is facilitated, weakening interactions between target compounds and the sample and thus improving mass transfer [17].

From a thermodynamic perspective, the temperature rise increases system entropy (Δ*S*), resulting in a more negative Gibbs free energy change (Δ*G* = Δ*H* − *T*Δ*S*) that thermodynamically favors analyte desorption and solubilization [18, 19]. Additionally, solvation plays a central role under these conditions. High temperatures can alter the solvent's polarity and dielectric constant, improving its ability to stabilize both polar and moderately nonpolar analytes.

The solvation process involves specific interactions between solvent molecules and analytes, forming solvation shells that enhance analyte diffusion into the liquid phase [18]. This synergy between improved solvation and favorable thermodynamic conditions accelerates the desorption–diffusion–solubilization sequence, underpinning the high extraction efficiency and reduced solvent consumption characteristic of EDGE.

In this study, EDGE demonstrated superior performance by achieving a 12.12% increase in phenolic yield from lemongrass leaf compared to other evaluated methods. This improvement is attributed to the synergistic action of moderate pressure (180 psi), which increases matrix permeability and promotes solvation, along with high temperature that reduces solvent viscosity and enhances diffusivity. Furthermore, the physicochemical properties of the extracted compounds likely favored improved recovery under the applied solvent conditions [19].

Besides its efficiency, EDGE is considered a sustainable alternative due to its shorter extraction time, lower energy and solvent consumption, and compatibility with automation and reproducibility [7, 17].

For CO, Alirezalu et al. [20] reported TPC values ranging from 19.98 to 62.08 mg GAE/g using ultrasound‐assisted extraction with methanol–water (80:20, 25 mL). Čulum et al. [21], investigating a *Crataegus* variety, obtained 14.43 mg GAE/g from leaves and flowers using Soxhlet with ethanol. Abu‐Gharbieh et al. [22] found 1.5 mg GAE/g in *Crataegus azarolus* leaves using maceration with 70% ethanol, whereas Ferioli et al. [23] reported only 0.056 mg GAE/g for *C. oxyacantha* leaves using ethanol–water (60:40, v/v) via maceration.

No data were found in the scientific literature regarding the total phenolic content of SB. This lack of information highlights the need for further investigation into this plant, which may have important implications for studies of its bioactive properties and applications in pharmacology and nutrition.

### 3.2 In Vitro Antioxidant Capacity

Various techniques have been employed to obtain antioxidant‐rich extracts from plant leaves [24]. This study assessed the antioxidant activity of the optimized extract using a single extraction cycle through ABTS, DPPH, and FRAP assays (Table 2).

**TABLE 2:** Results for antioxidant capacity of the analyzed extracts (mean ± standard deviation).

Assay	CC	SB	CO
^a^ABTS	55.71 ± 2.74	19.5 ± 0.92	82.41 ± 4.14
^a^DPPH	22.34 ± 0.10	5.53 ± 0.55	54.64 ± 0.27
^b^FRAP	1,158.92 ± 29.87	1,216.3 ± 47.02	1,920.4 ± 66.39

Abbreviation: CC, *Cymbopogon citratus*; CO, *Crataegus oxyacantha*; DPPH, 2,2‐diphenyl‐1‐picrylhydrazyl; FRAP, ferric reducing antioxidant power; SB, *Sorocea bonplandii*.

^a^ Results of ABTS and DPPH are expressed in mg TE/g of leaves (in dray basis).

^b^ Results of FRAP is expressed in µM Fe^2+^g^−1^ of leaves (in dry basis).

The ABTS assay showed that, under optimal extraction conditions, the radical scavenging activity was 55.71 ± 2.74, 19.50 ± 0.92, and 82.41 ± 4.14 mg TE/g for the CC, SB, and CO samples, respectively. This assay is based on the reaction with the ABTS^+^• organic cation radical, and its mechanism may involve either hydrogen atom transfer (HAT) or a single electron transfer (SET) [24].

The antioxidant activity measured by the DPPH assay reflects the electron‐donating ability of compounds present in the samples [24]. Antioxidant activities observed for CC, SB, and CO were 22.34 ± 0.10, 5.53 ± 0.55, and 54.64 ± 0.27 mg TE/g, respectively.

The antioxidant capacity in crude optimized extracts was evaluated using the FRAP assay. The antioxidant activity of the species CO, SB, and CC Stapff Showed greater reducing potential, with antioxidant activities of 1920.42 ± 66.39, 1216.32 ± 47.02, and 1158.92 ± 29.87 mg TE/g, respectively. The antioxidant activity of hydroethanolic extracts measured by FRAP method was higher than the other antioxidant assays due to its specificity for hydrophilic phenolic antioxidants. In comparison, ABTS and DPPH assays detect both hydrophilic and lipophilic antioxidants, although with varying affinities [24].

Several studies have reported antioxidant activity in *Crataegus* species using different extraction methods, assays, and analytical standards. Belabdelli et al. [25] reported 22 mg VCE/g (ascorbic acid equivalents per gram of dry weight) in ethanolic leaf extracts using the DPPH assay. Ferioli et al. [23], applying the ABTS assay to hydroethanolic extracts, observed values ranging from 31.48 to 52.58 mg TE/g DW. Hamza et al. [26] reported 877.8 µmol VCE/g in extracts from aerial parts of white hawthorn evaluated by the FRAP assay, corresponding to approximately 154.5 mg VCE/g. In the present study, CO showed antioxidant activity of 82.41 ± 4.14 mg TE/g (ABTS), 54.64 ± 0.27 mg TE/g (DPPH), and 1920.4 ± 66.39 µmol Fe^2+^/g (FRAP). Although different standards were used (Fe^2+^, ascorbic acid, and Trolox), these values indicate strong antioxidant potential, comparable to the levels obtained in the present study expressed as mg TE/g. Direct numerical comparison should be interpreted cautiously due to methodological and standard differences.

The methodologies applied to evaluate the scavenging capacity of the optimized extracts yielded consistent results. The combined use of these assays provided a more comprehensive and accurate assessment of the in vitro antioxidant profile of the plant materials.

Each method evaluates a different aspect of antioxidant action, and the integration of results enables a more robust and reliable characterization.

### 3.3 Quantitative Analysis of Phenolic Compounds by HPLC‐PDA

Samples were filtered through a 20 µm nitrile membrane and analyzed by HPLC‐PDA using gradient elution on a reversed‐phase column (total runtime: 70 min). The mobile phase consisted of methanol and 5% (v/v) acetic acid in water.

HPLC‐PDA enabled reliable identification and quantification of key analytes using certified standards under identical conditions. Compound identity was confirmed by retention time and *λ*_max_ comparison with reference standards. Quantification was based on calibration curves ranging from 1 to 50 mg/L for caffeic, *p*‐coumaric, ferulic, and cinnamic acids and theophylline; for rutin, an additional range (50–150 mg/L) was applied.

The method was validated for linearity (*R* ^2^  ≥  0.99), limit of detection (LOD), limit of quantification (LOQ), precision, and accuracy. LOD and LOQ were calculated from calibration curve statistics as described by Igbokwe et al. [27]. Full validation data are provided in the Supporting Information section.

**FIGURE 2:**
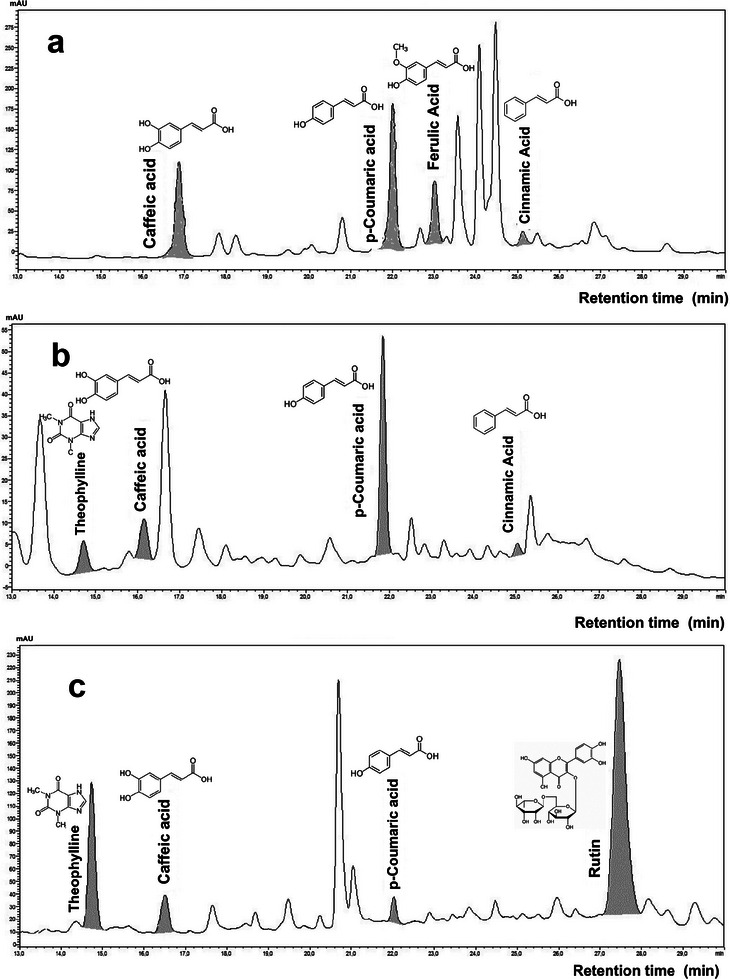
Chromatograms obtained for each sample according to their specific wavelengths. (a) CC; (b) SB; and (c) CO. Chromatographic conditions are described in Section 2.

Analytical curves confirmed linearity across the specified ranges. Chromatographic profiles are shown in Figure 2, and quantified phenolic compounds are summarized in Table 3.

**TABLE 3:** Content of phenolic compounds identified in herbal leaf extracts.

	Concentration (µg/L) ± SD			Merit figures		
Compounds	CC	SB	CO	LOD mg/L	LOQ mg/L	Recovery (%) (*n* = 3)
Caffeic acid	15.01 ± 0.12	14.59 ± 0.07	10.98 ± 0.01	0.07	0.22	103
*p*‐Coumaric acid	3.7 ± 0.04	3.49 ± 0.20	5.78 ± 0.01	0.08	0.25	101
Ferulic acid	16.49 ± 0.1	n.d.	n.d.	0.28	0.38	94
Cinnamic acid	3.06 ± 0.01	3.82 ± 0.32	n.d.	0.07	0.22	102
Theophylline	n.d.	2.49 ± 0.05	6.37 ± 0.02	0.07	0.21	102
Rutin	n.d.	n.d.	93.05 ± 0.27	0.09	0.28	101

*Note*: Data are expressed as mean values from three experiments (±SD).

Abbreviations: LOD, limits of detection; n.d., not detected; SD, standard deviation.

Phenolic acids, a methylxanthine, and a flavonol were consistently identified in the analyzed herbal samples, based on comparison with certified standards. In the CC extract, phenolic acids predominated, with notable concentrations of caffeic (15.01 ± 0.12 µg/L), *p*‐coumaric (3.70 ± 0.04 µg/L), ferulic (16.49 ± 0.10 µg/L), and *trans*‐cinnamic acid (3.06 ± 0.01 µg/L). The CO extract showed a more diverse profile, including caffeic (10.98 ± 0.01 µg/L), *p*‐coumaric acid (5.78 ± 0.01 µg/L), the flavonol rutin (93.05 ± 0.27 µg/L), and the methylxanthine theophylline (6.37 ± 0.02 µg/L). In the SB extract, caffeic (14.59 ± 0.07 µg/L), *p*‐coumaric (3.49 ± 0.20 µg/L), *trans*‐cinnamic acid (3.82 ± 0.32 µg/L), and theophylline (2.49 ± 0.05 µg/L) were present. These findings are consistent with prior studies confirming the presence of phenolic acids in CC and *C. oxyacantha* [28, 29].

The occurrence of caffeic and *p*‐coumaric acids across all samples underscores their relevance as core contributors to antioxidant activity, given their widely recognized antioxidant, anti‐inflammatory, and antimicrobial effects [28, 29]. Among these, cinnamic acid is particularly effective in stabilizing free radicals due to its conjugated double bond, which facilitates resonance stabilization [28, 29].

Rutin stood out in this study, being detected exclusively in the CO sample at a notably high concentration (93.05 ± 0.27 µg/L). This result aligns with the literature, where rutin is closely associated with the pharmacological potential of *Crataegus* species [30]. Known for its broad biological activity, including antioxidant, anti‐inflammatory, vasoprotective, and cardiotonic effects, rutin has drawn attention in studies targeting cardiovascular health and oxidative stress [31]. However, its effectiveness depends not only on concentration but also on bioavailability, which is influenced by glycosylation, intestinal absorption, and metabolism. According to Frutos et al. [32], despite being abundant in plant matrices, the biological action of rutin is shaped by its ability to reach systemic circulation and target tissues. Therefore, the elevated levels observed in the CO extract may offer therapeutic relevance, provided that adequate bioavailability is achieved in vivo.

Theophylline, detected in both SB and CO extracts, is a methylated xanthine recognized for its antioxidant and bronchodilator activities [33]. Its concentrations in SB and CO samples, 2.49 ± 0.05 and 6.37 ± 0.02 µg/L, respectively, suggest a possible contribution to the pharmacological and antioxidant profile of these extracts. As a naturally occurring compound in teas and infusions, its presence reinforces the bioactive potential of these plants.

Lastly, our findings are supported by previous chromatographic studies. Coelho et al. [34] confirmed the presence of caffeic, ferulic, and *p*‐coumaric acids in CC, whereas Muala et al. [35] reported caffeic, *p*‐coumaric, ferulic, and cinnamic acids in similar matrices, alongside vanillic, gallic, syringic, and dihydroxybenzoic acids—several of which were also indicated by unconfirmed peaks in our profiles.

Other compounds were observed in samples; however, there no positive identification based on the certified standards of phenolic compounds used in the development of chromatographic methodology.

### 3.4 Relationship Among Total Phenolic Content, Extract Composition, and In Vitro Antioxidant Activity

**FIGURE 3:**
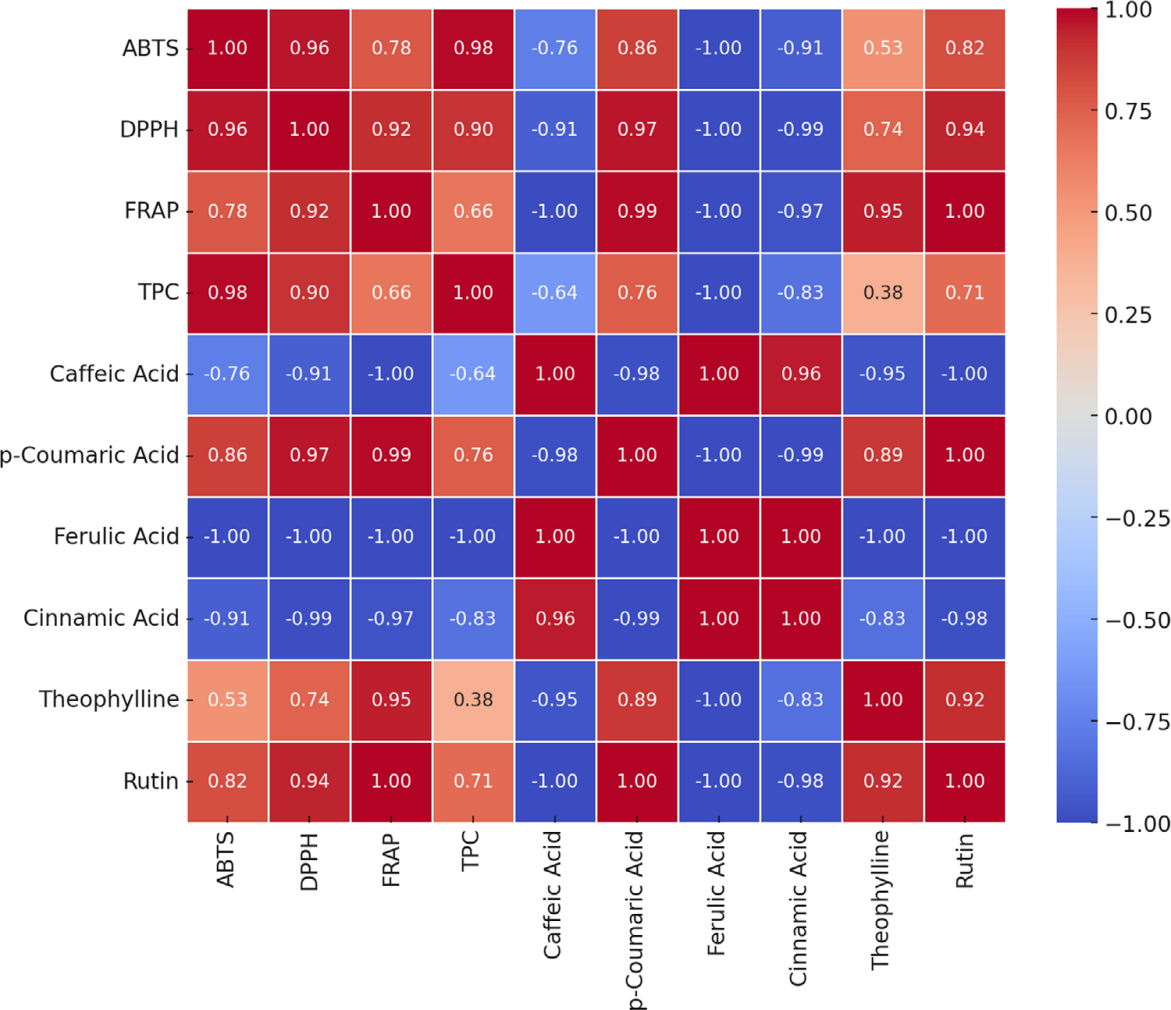
Correlation matrix of interactions among antioxidant activities, total phenolic content, and composition of herbal extracts. DPPH, 2,2‐diphenyl‐1‐picrylhydrazyl; FRAP, ferric reducing antioxidant power.

The relationship between antioxidant activity and total phenolic compound (TPC) content is essential for understanding the biochemical interactions that influence the antioxidant capacity of natural matrices. To illustrate these interactions, a correlation matrix (Figure 3) was used, providing a clear visualization of both positive and negative associations among the evaluated variables [36]. This approach clarifies how individual phenolic compounds contribute to antioxidant activity and offers insight into potential synergistic or antagonistic effects within the analyzed matrix.

Figure 3 illustrates the correlation trends between antioxidant capacity assays and the phenolic content of extracts obtained using the EDGE technique. TPC exhibited a strong positive correlation with ABTS (*r* = 0.98) and DPPH (*r* = 0.90) and a moderate positive correlation with FRAP (*r* = 0.66), indicating that higher levels of phenolic compounds are associated with greater antioxidant activity [36]. These results emphasize the essential role of phenolic compounds in free radical scavenging and iron‐reducing mechanisms.

Given their relevance, the efficiency of phenolic compound extraction becomes a critical factor, particularly when optimizing antioxidant responses. The efficient extraction of bioactive compounds from plant matrices depends on selecting appropriated solvents and extraction methods [37]. Solvent choice is primarily based on polarity, which directly influences the solubility of target analytes based on their structural and physicochemical properties. Water and ethanol, due to their favorable polarity profiles, are commonly employed in bioprospecting and have shown high efficiency in the extraction of phenolic compounds [37]. In contrast, less polar solvents yield lower phenolic content, reducing the extract's free radical scavenging capacity [38].

This study also investigated the correlation between antioxidant assays and the individual phenolic composition of the extracts optimized through EDGE. Phenolic acids, particularly caffeic and *p*‐coumaric acids, significantly contributed to the total phenolic compound (TPC), reinforcing their key role in antioxidant activity. The strong correlation observed between these acids and the antioxidant capacity across all analyzed samples further supports this conclusion.

Rutin exhibited a moderate correlation due to its exclusive presence in the CO sample, where it was strongly associated with FRAP assay values. In contrast, theophylline, identified in the SB and CO samples, displayed a weak correlation with TPC, as it is not a phenolic compound, limiting its contribution to antioxidant activity.

Flavonoid solubility depends on polarity: hydroxylated structures dissolve in polar solvents, whereas methoxylated ones need nonpolar solvents. As hydroxyl groups enhance antioxidant activity, polar extracts like CO—rich in water‐soluble rutin—show strong FRAP responses [38]. However, rutin is thermosensitive and degrades above 100°C, breaking down into simpler compounds [39].

Phenolic acids are highly soluble in ethanol–water mixtures, with solubility influenced by solvent composition [40]. The 40:60 (v/v) ethanol–water ratio yielded high phenolic content in all samples. This correlates with the ABTS assay, which detects both hydrophilic and lipophilic antioxidants [41], whereas the DPPH assay, more selective for hydrophobic compounds, showed lower activity, indicating a limited presence of such analytes [38, 39, 40].

TPC includes structurally diverse compounds with varying physicochemical properties. This study relates TPC to antioxidant activity by considering log *K*_ow_ and p*K*a values (see Supporting Information section) [40, 42].

The CC sample showed abundant phenolic acids and unidentified polar compounds. Due to their low log *K*_ow_, phenolic acids are highly water‐soluble and show low bioaccumulation [41]. Similarly, CO and SB samples contained phenolic acids, a methylxanthine, and a flavonol, all hydrophilic, supporting their strong ABTS and FRAP activities.

Antioxidant trends reflected compound polarity: low DPPH values suggest fewer hydrophobic analytes, whereas stronger ABTS/FRAP responses indicate a predominance of hydrophilic constituents, consistent with the water‐rich solvent system [41, 42, 43].

The p*K*a values influence solubility, redox potential, and antioxidant reactivity by modulating compound ionization at specific pH levels [43]. These properties affect extraction efficiency and are key in predicting pharmaceutical behavior and designing green extraction strategies [43].

Significant differences emerged in the correlations among TPC, extract composition, and antioxidant assay results. The CO sample exhibited the highest antioxidant activity across all assays, reinforcing the direct relationship between phenolic content and antioxidant capacity. However, individual composition influenced these values. For example, the CC sample demonstrated strong antioxidant activity despite intermediate TPC, suggesting that the compound quality (e.g., phenolic acids) is as critical as quantity. The SB sample had the lowest TPC but still showed moderate antioxidant activity, though lower than other samples. The presence of unidentified compounds in SB and other extracts suggests potential contributions to antioxidant behavior that warrant further investigation.

## 4 Conclusions

This study identified optimal EDGE conditions for extracting antioxidant phenolics: 40% ethanol, 60% water at 165.8°C for 6.03 min. The model was significant (*p* < 0.05), with *R* ^2^ = 0.99 and a midpoint of 69.18% ± 0.73%. EDGE proved efficient, rapid, and reproducible, reducing solvent use and simplifying sample preparation, while yielding extracts rich in phenolic compounds with antioxidant potential.

Chromatographic analysis confirmed the presence of phenolic acids in all samples, with theophylline detected in SB and CO and high rutin content in CO. Other compounds remained unidentified. These compounds correlated with TPC values and antioxidant activity, supporting their contribution to extract bioactivity. Detailed characterization of these constituents is essential to elucidate their bioactivity and therapeutic potential, contributing to extract quality assessment.

## Conflicts of Interest

The authors declare no conflicts of interest.

## Supporting information




**Supporting File 1**: jssc70250‐sup‐0001‐SuppMat.pdf.

## Data Availability

The authors have nothing to report.
