# Supplementary material for: Enhanced Energized Dispersive–Guided Extraction Using Doehlert Matrix Optimization and Antioxidant Evaluation of Phenolic Compounds From Herbal Leaves by Using High‐Performance Liquid Chromatography
Source: J Sep Sci. 2025 Sep 10;48(9):e70250. doi: 10.1002/jssc.70250 (PMC12423361; doi:10.1002/jssc.70250)
Supplement: Supplementary file 1 — Supporting File 1: jssc70250‐sup‐0001‐SuppMat.pdf. [file JSSC-48-e70250-s001.pdf]

Supplementary material

Title: Enhanced Energized Dispersion Guided Extraction Using Doehlert Matrix Optimization and Antioxidant Evaluation of Phenolic Compounds from Herbal Leaves by using High Performance Liquid Chromatography

Authors:

Rosianne P. Silva[a], Paulo N. A. Santos[b], Luciana L. Nascimento[c], Alini T. Fricks[c], Allan S. Polidoro[d], Elina B. Caramão [a, b, e]*

Affiliations:

[a]*Programa de Pós-Graduação em Química, Universidade Federal de Sergipe, São Cristóvão, SE, 49680-000, Brazil;*

[b]*Rede Nordeste de Biotecnologia, Universidade Federal de Sergipe, São Cristóvão, SE 49100-000, Brazil;*

[c]*Departamento de Análise Bromatológica, Programa de Pós-Graduação em Ciência de Alimentos (PGALI), Faculdade de Farmácia, Universidade Federal da* 13 *Bahia, Salvador 40170-115, Brazil;*

[d]*Department of Chemistry, Pharmaceutical, and Agricultural Sciences, University of Ferrara, Ferrara, Italy.*

[e]*Instituto Nacional de Ciência e Tecnologia, Energia e Ambiente (INCT E&A), Salvador, BA, Brazil.*

(*) to whom correspondence should be addressed – elina@ufrgs.br

Table of Contents (in order of appearance in SM):

Figure S1. Contour diagram representing the extraction yield of total phenolic compounds as a function of temperature and extraction time, at a fixed ethanol concentration of 60%. The model was generated based on the experimental design described in the Methods Section.

Table S1. Coded and real values used in Doehlert's Design

Table S2. Results of ANOVA for the optimization of TPC in lemongrass (CC).

Table S3. Data from the construction of the analytical curve and validation for the determination of phenolic compounds by HPLC/PDA.

Figure S2. Representative chromatogram of the standard mixture analyzed at a concentration of 20 ppm. Chromatographic conditions are described in the Methods section.

Table S4. Standards used in the mix of phenolic compounds, retention times and maximum absorption wavelengths, molecular mass, molecular formula, acidity constant and octanol-water partition coefficient.

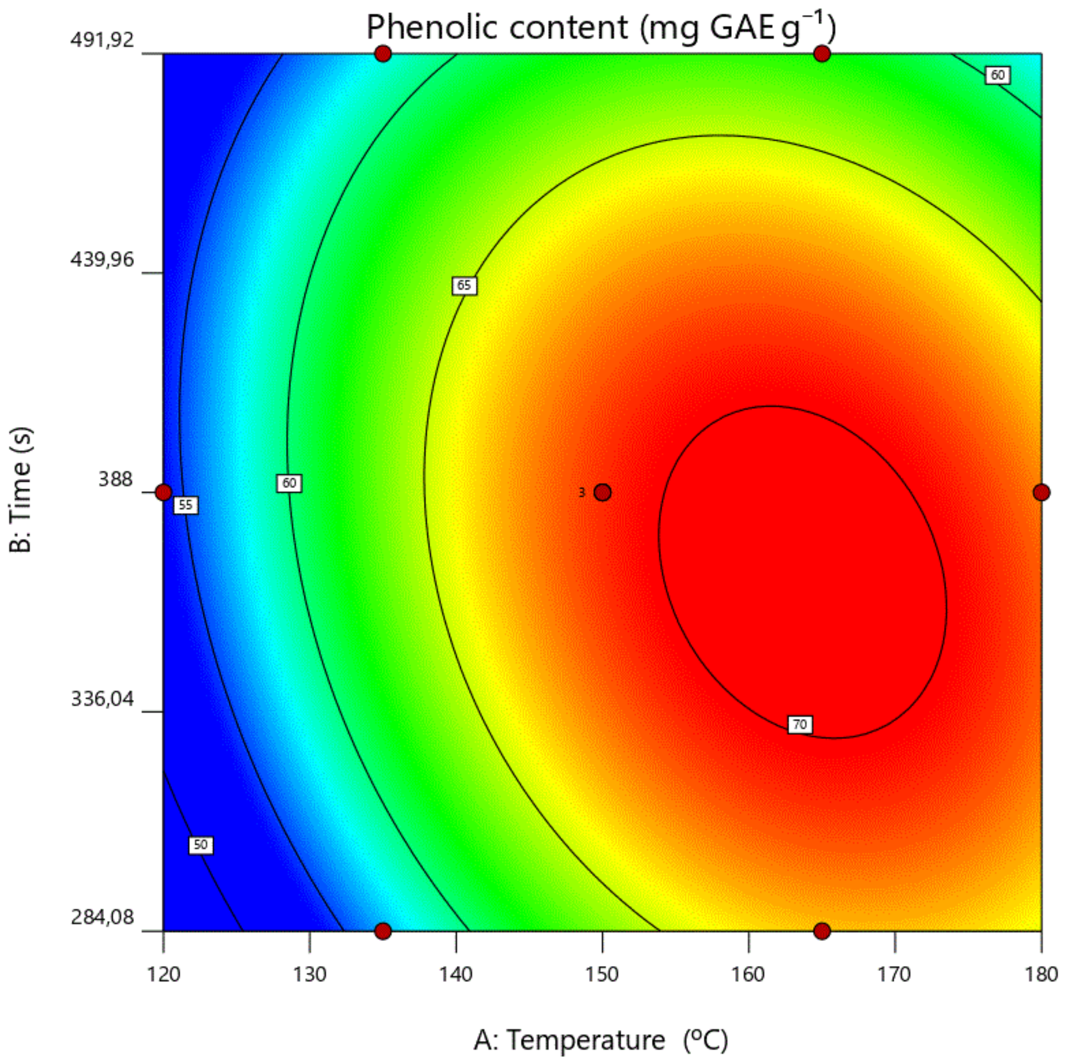

Figure S1. Contour diagram representing the extraction yield of total phenolic compounds as a function of temperature and extraction time, at a fixed ethanol concentration of 60%. The model was generated based on the experimental design described in the Methods Section.

Table S1. Coded and real values used in Doehlert's Design

Experiment	Temperature (°C) (x_1)	Time (min) (x_2)
1	1 (180)	0 (6,47)
2	0.5 (165)	0.866 (8,20)
3	-1 (120)	0 (6,47)
4	-0.5 (135)	-0.866 (4,74)
5	0.5 (165)	-0.866 (4,74)
6	-0.5 (135)	0.866 (8,20)
7 (C)	0 (150)	0 (6,47)
8 (C)	0 (150)	0 (6,47)
9 (C)	0 (150)	0 (6,47)

Table S2: Results of ANOVA for the optimization of TPC in lemongrass (CC)

**Parameters**	**Quadratic Sum (QS)**	**GL**	**Quadratic Average (QA)**	F_{calc}	***p*-value**
Model	294.58	5	58.92	108.93	0.0014
Temperature (x_1)	145.53	1	145.53	269.07	0.0005
Time (x_1)	2.71	1	2.71	5.00	0.1113
(x_1*x_2)	9.83	1	9.83	18.17	0.0237
$(x_1)^2$	81.94	1	81.94	151.49	0.0012
$(x_2)^2$	81.87	1	81.87	151.37	0.0012
Lack of Adjustment	0.0038	1	0.0038	0.0046	0.9519
Pure Error	1.62	2	0.8094		

R^2 = 0.99

Table S3. Data from the construction of the analytical curve for the determination of phenolic compounds by HPLC/PDA.

Compounds	T_R (min)	$\lambda_{máx.}$ (nm)	Equation	Linearity (R^2)	Working rang (mg L^{-1})	LOD mg L^{-1}	LOQ mg L^{-1}	Recovery (%) (n=3)
**Caffeic acid**	16.79	322	y= 9. 10^{-6} x + 2.1	0.9970	1 a 50	0.07	0.22	103
**p-Coumaric ac**	21.98	310	y= 5. 10^{-6} x + 2.1	0.9963	1 a 50	0.08	0.25	101
**Ferulic acid**	23.54	240	y= 8. 10^{-6} x + 3.7	0.9900	1 a 50	0.28	0.38	94
**Cinnamic acid**	25.24	280	y= 1. 10^{-5} x + 2.0	0.9970	1 a 50	0.07	0.22	102
**Theophylline**	14.76	275	y= 6. 10^{-6} x + 2.2	0.9969	1 a 50	0.07	0.21	102
**Rutin** curve 1	27.19	255	y= 9. 10^{-5} x + 2.6	0.9962	1 a 50	0.09	0.28	-
**Rutin** curve 2	27.19	255	y= 2. 10^{-5} x + 15.	0.9943	50 a 150	0.09	0.28	101

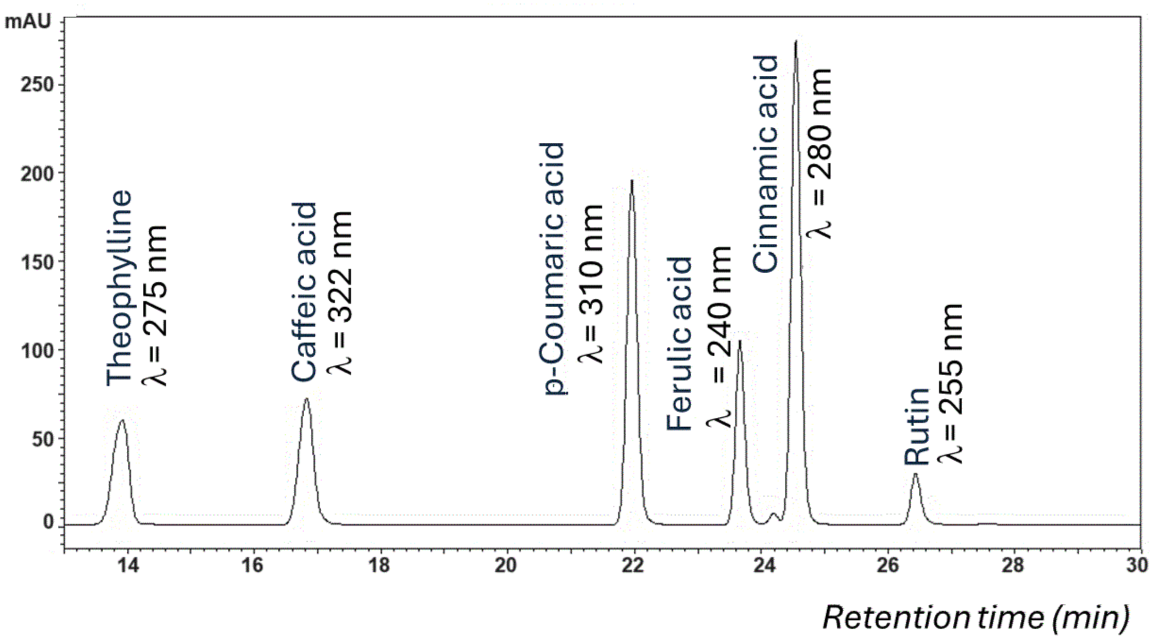

Figure S2. Representative chromatogram of the standard mixture analyzed at a concentration of 20 ppm. Chromatographic conditions are described in the Methods section.

Table S4. Standards used in the mix of phenolic compounds, retention times and maximum absorption wavelengths, molecular mass, molecular formula, acidity constant and octanol-water partition coefficient.

Phenolic compounds	Class	Molecular formula	PM (g mol^{-1})	pKa	Log K_{ow}	T_R (min)	$\lambda_{máx.}$(nm)
Caffeic acid	Phenolic acid	$C_9H_8O_4$	180,16	4,62	1,15	16,79	322
p-Coumaric acid	Phenolic acid	$C_9H_8O_3$	164,16	4,64;9,45	1,79	21,98	310
Cinnamic acid	Phenolic acid	$C_9H_8O_2$	148,16	4,45	2,07	25,24	280
Ferulic acid	Phenolic acid	$C_{10}H_{10}O_4$	194,18	4,58	1,51	23,54	240
Theophylline	Alkaloid	$C_7H_8N_4O_2$	180,16	8,81	-0,02	14,76	275
Rutin	Flavonoid	$C_{27}H_{30}O_{16}$	650,52	-	-	27,19	255
